# Intestinal Levodopa/Carbidopa Infusion as a Therapeutic Option for Unresponsive Freezing of Gait after Deep Brain Stimulation in Parkinson's Disease

**DOI:** 10.1155/2020/1627264

**Published:** 2020-05-14

**Authors:** Belén González-Herrero, Serge Jauma-Classen, Roser Gómez-Llopico, Gerard Plans, Matilde Calopa

**Affiliations:** Bellvitge University Hospital-Biomedical Research Institute (IDIBELL), L'Hospitalet de Llobregat, Barcelona, Spain

## Abstract

**Background:**

Treatment of freezing of gait (FOG) is always challenging because of its unpredictable nature and multifactorial physiopathology. Intestinal levodopa infusion has been proposed in recent years as a valuable option for its improvement. FOG in Parkinson's disease (PD) can appear after deep brain stimulation in patients who never had gait symptoms.

**Objective:**

To study the effects of intestinal levodopa/carbidopa infusion in unresponsive-FOG that appears in PD patients treated with subthalamic nucleus deep brain stimulation.

**Methods:**

We retrospectively collected and analyzed demographic, clinical, and therapeutic data from five PD patients treated with subthalamic nucleus stimulation who developed unresponsive-FOG and received intestinal levodopa/carbidopa infusion as an alternative therapy. FOG was measured based on scores in item 14 of the Unified Parkinson's Disease Rating Scale before and after intestinal levodopa infusion.

**Results:**

Administration of intestinal levodopa caused improvement of FOG in the “ON” state in four patients (80%) by 2 or more points in item 14 of the Unified Parkinson's Disease Rating Scale. The improvement was maintained for at least 12 months.

**Conclusions:**

Intestinal levodopa infusion may be a valuable therapeutic option for unresponsive-FOG developed after subthalamic nucleus deep brain stimulation.

## 1. Introduction

Freezing of gait (FOG) is defined as short episodes of the inability to initiate or continue stepping forward and is typically more evident when turning, facing a narrow space, or during stress and distraction. Generally, it is related to the duration and severity of Parkinson's disease (PD) and is influenced by sensory and cognitive inputs; however, uncertainty remains over its ultimate cause [1]. Based on the response to dopaminergic medication, four types of FOG have been characterized: OFF-type-FOG, pseudo-ON-FOG, unresponsive-FOG, and true FOG-ON [2].

Treatment of FOG is a clinical challenge, and different strategies such as modification of levodopa dosage, adding catechol-O-methyltransferase inhibitors or amantadine, deep brain stimulation (DBS), apomorphine, metilphenidate, and even electroconvulsive therapy have been used to improve this phenomenon with varying outcomes [3]. DBS has shown significant improvement in gait and FOG [4] but in some PD patients can aggravate or even induce FOG and postural instability [5–7]. Previous case reports [8, 9] and two recent studies [10, 11] have found that FOG resistant to conventional oral therapy may benefit from levodopa/carbidopa intestinal gel (LCIG) infusion.

Based on this information, we evaluated the effects of LCIG in unresponsive-FOG with special interest in the ON state (FOG-ON) that appears *de novo* after subthalamic nucleus deep brain stimulation (STN-DBS) therapy in PD patients.

## 2. Patients and Methods

Among the 48 PD patients treated with STN-DBS in Bellvitge University Hospital (Barcelona, Spain) between 2010 and 2018, 5 patients (P1, P2, P3, P4, and P5) developed unresponsive-FOG with no previous history of FOG.

FOG is considered “unresponsive” by the presence of FOG in both the OFF and ON states and is not influenced by medication [2]. In our study, we considered FOG to be unresponsive after trying all possible adjustments of DBS (including switching OFF) and combinations of oral medication.

The five patients were selected for treatment with LCIG as an alternative advanced therapy. DBS was switched OFF in all patients at least 24 h prior to starting LCIG to better assess the response to intestinal levodopa infusion. LCIG infusion via jejunostomy was started according to expert guidelines, following a nasoduodenal trial.

Demographic, clinical details, DBS state, and characteristics of treatment with LCIG in the five patients were retrospectively collected (Tables 1 and 2). FOG-ON scores based on item 14 in the Unified Parkinson's Disease Rating Scale (UPDRS) were determined in four treatment conditions: (i) DBS ON and oral medication (ON DBS–ON Med), (ii) only oral medication (OFF DBS–ON Med), (iii) after 1 month of LCIG treatment, and (iv) after 12 months of LCIG treatment.

FOG assessment was carried out an hour after the first morning oral dose of L-Dopa and in daily ON condition during LCIG infusion.

## 3. Results

### 3.1. Patients' Clinical, Demographic, and DBS Data

In total, 5 of the 48 patients treated with STN-DBS between 2010 and 2018 developed unresponsive-FOG after surgery. All of the patients were women with a median age of 66 (57–69) years at the time of surgery, and a median age of 11 (6–12) years since disease onset. None of them had cognitive impairment. The appearance of FOG was not related to any surgery complication. The median period of time between surgery and appearance of unresponsive-FOG was 10 (2.5–48) months. P3 suffered an infection in the left electrode after 2.5 months, but it was correctly replaced and she remained without gait disturbances until the 10^th^ month after the first surgery. For P1 and P2, DBS was switched OFF 2 months before LCIG, and they were only treated with oral medication because, in addition to the unresponsive-FOG, STN stimulation did not achieve a fully satisfactory control of motor fluctuations. To check the correct positioning of the electrodes within the STN in these two patients, stereotactic coordinates of the center of each electrode were referenced to the mid-commissural point. In P1, the right electrode was 11.5 (X), −3.5 (Y), and −3.75 (Z), and the left electrode was 11.5 (X), −5 (Y), and −2.5 (Z). In P2, the right electrode was 13.14 (X), −3.5 (Y), and −2.15 (Z), and the left electrode was 11.5 (X), −3.58 (Y), and −2.97 (Z). For P3, P4, and P5, DBS continued ON bilaterally until 24 h prior to starting LCIG because, despite FOG, STN-DBS showed significant clinical benefits. These data are shown in Table 1.

### 3.2. Characteristics of Treatment with LCIG Infusion and FOG Data

The median age at the beginning of LCIG was 66 (59–71) years, and the median time between surgery and initiation of LCIG was 18 (8–36) months. Four patients (80%, P1–P4) had improvement in the FOG-ON with LCIG equal to or greater than 2 points on item 14 in the UPDRS scale measured at the first month follow-up. The improvement was maintained in the four patients for at least 12 months (videos pre/post-LCIG of P1 and P3 are provided). In P1, P2, and P3, an important improvement in troublesome dyskinesias with LCIG was also seen. Subsequently, two of them (P3 and P4) needed dual therapy (DBS-LCIG) for better control of the overall PD symptoms. In P5, FOG-ON did not improve and LCIG was discontinued after 1-month follow-up, and the patient returned to only DBS ON.

These data and the total equivalent doses of levodopa in each situation are shown in Tables 2 and 3.

## 4. Discussion

LCIG is available for the treatment of advanced PD with severe motor fluctuations and dyskinesias that are no longer controlled by first-line therapies. Since its first use reported in 1993, LCIG has become a valuable option for advanced PD control and more indications including nonmotor symptoms are being studied with promising results [12].

Worsening of gait after DBS has been reported in PD and non-PD patients [7]. This deterioration may happen within the first days or weeks following DBS surgery and resolve itself with time (microlesioning effect of the surgery) or after adjustments in stimulator parameters and oral medication [13]. However, gait problems may persist in a proportion of patients regardless of therapy settings [5–7].

In our cohort of 48 PD operated patients between 2010 and 2018, 10% developed unresponsive-FOG *de novo*. Buhmann et al. [7] found that about 12% of STN-stimulated PD patients exhibited nonreversible gait disturbances within the first 6 months of STN stimulation. The cause of this worsening of gait after DBS is still not fully understood. Incorrect positioning of the electrodes [14, 15], diffusion of the stimulatory field to the pedunculopontine nucleus pathway, or overactivity in STN, which leads to inhibitory input via internal globus pallidus to the final common pathway of the brainstem locomotor regions that control gait [16, 17], are some theories for this deterioration. We consider the fact that FOG did not improve after a minimum of 24 h DBS-OFF in any of our patients that it is unlikely that the gait problems were directly related to stimulation.

It has also been suggested that deterioration in gait is more likely related to surgery if FOG appears within 6 months after the procedure [7]. In our study, with the exception of P5, resistant FOG-ON appeared at least 6 months after surgery.

FOG has always been a challenging symptom in terms of therapeutic approaches. Chang et al. [8] reported the benefits of 24 h LCIG for the treatment of five PD patients with unresponsive-FOG. In a recent longitudinal study, Rispoli et al. [11] evaluated the effectiveness of LCIG on balance and gait, with particular attention being given to FOG in 15 patients with advanced PD. The authors found significant improvement with LCIG in all patients not related to overall total equivalent dose of levodopa (LED), but most likely related to the fine tailoring of LCIG.

Regidor et al. [18] published findings from a study of 19 patients, which suggested that dual therapy (LCIG-DBS) may be an option for treating advanced PD patients who develop refractory symptoms after years of acceptable clinical control with DBS. They divided those symptoms into three groups: axial symptoms measured by the UPDRS axial subscale, severe pain, and dystonia. They found significant improvement in the UPDRS axial subscale with DBS-LCIG; however, they did not specify which item in the axial subscale improved.

In our patients, one reason for improvement of the unresponsive-FOG could be related to the tolerance of a higher LED with LCIG as seen in P3 and P4. This is in accordance with the hypothesis that most FOG during the ON state may actually be pseudo-ON-FOG and represent patients who are relatively undertreated, because the “threshold” to improve FOG is higher than that to improve appendicular motor signs [19]. However, in P1 and P2, the dose remained almost unchanged (Table 2). Therefore, we assume that the benefit is not only related to a higher LED, but also to the more stable plasma level that continuous intestinal delivery provides, in accordance with the study by Rispoli et al. [11].

To note, LCIG helped control troublesome dyskinesias in P1, P2, and P3. One may also hypothesize that troublesome dyskinesia played a role in the poor gait performance [20].

The study has limitations including a small sample size, the fact data was collected retrospectively, and that FOG was only measured using the UPDRS item 14. The relatively short follow-up may also be considered a limitation because, according to a recently published study [21], control over axial symptoms seems to diminish after 4 years of initiating LCIG.

## 5. Conclusions

LCIG might be a valuable therapeutic option for unresponsive-FOG after STN-DBS. The improvement of FOG-ON and overall satisfaction of our patients after LCIG were notable. Therefore, future larger trials should be conducted to confirm these findings.

## Figures and Tables

**Table 1:** Clinical, demographic, and surgical data.

Patient	Sex	Age at the time of STN-DBS (years)^*∗*^	Time from diagnosis to STN-DBS (years)^*∗∗*^	Latency between surgery and unresponsive-FOG onset (months)^*∗∗∗*^	Mattis Dementia rating scale	Surgery complications	DBS ON/OFF until 24 h before LCIG	Daily time spent in OFF before LCIG (UPDRS item 39)	Functional impact of dyskinesias before LCIG (UPDRS item 33)
P1	F	67	11	20	Normal	No	OFF	3	2
P2	F	69	6	8	Normal	No	OFF	3	2
P3	F	57	11	10	Normal	Infection left electrode	ON	3	3
P4	F	64	10	48	Normal	No	ON	2	1
P5	F	66	12	2.5	Normal	No	ON	3	2

^*∗*^Median age (years) at the time of STN-DBS: 66 IQR (57–69). ^*∗∗*^Median time (years) from diagnosis to STN-DBS: 11 IQR (6–12). ^*∗∗∗*^Median latency (months) between surgery and unresponsive-FOG onset: 10 IQR (2.5–48). F = female; IQR = interquartile range.

**Table 2:** Characteristic of the treatment with LCIG and levodopa-equivalent doses in the five patients.

Patient	Age at the time of LCIG (years)^*∗*^	LED pre-LCIG (mg)^*∗∗*^	LED post-LCIG (LCIG + CR) 1st month (mg)^*∗∗∗*^	LED post-LCIG (LCIG + CR) 12 months (mg)^*∗∗∗∗*^	DBS ON/OFF at 12 months	Daily time spent in OFF after LCIG (UPDRS Item 39)	Functional impact of dyskinesias after LCIG (UPDRS item 33)
P1	71	2250	2164	2310	OFF	1	1
P2	70	2400	2130	1960	OFF	1	1
P3	59	855	1233	1098	ON	1	1
P4	70	885	1638	1914	ON	1	1
P5	66	1874	2100	NA	ON	NA	NA

^*∗*^Median age (years) at the time of LCIG: 66 IQR (59–71). ^*∗∗*^Median LED (mg) pre-LCIG: 1874 IQR (855–2400). ^*∗∗∗*^Median LED (mg) post-LCIG after 1 month: 2100 IQR (1233–2164). ^*∗∗∗∗*^Median LED (mg) post-LCIG after 12 months: 1937 IQR (1098–2310). LED pre-LCIG = total equivalent dose of levodopa oral medication; LED post-LCIG = total equivalent dose of levodopa after LCIG (LCIG plus oral control release); NA = not applicable; IQR = interquartile range.

**Table 3:** Changes in FOG-ON based on item 14 in the UPDRS scale in the different scenarios.

FOG-ON pre-LCIG		FOG-ON 1st month post-LCIG	FOG-ON 12 months post-LCIG
ON DBS–ON Med	OFF DBS–ON Med		
3	3	1	1
3	3	0	1
4	4	1	1
4	4	2	2
4	4	3	NA

ON DBS–ON Med = DBS ON and oral medication; OFF DBS–ON Med = Only oral medication.

## Data Availability

The study data are available on request to the corresponding author.

## Additional Points

*Highlights*. Freezing of gait in Parkinson's disease can appear after deep brain stimulation. Treatment of freezing of gait is challenging. Intestinal levodopa/carbidopa could be a therapeutic option in this situation. The improvement might be related with higher dose and more stable plasma levels with intestinal levodopa/carbidopa.

## Supplementary Materials

Supplementary Materials Video 1 (P1 pre-LCIG): freezing of gait in the ON state in patient 1 before treatment with levodopa/carbidopa intestinal gel. This clip shows patient 1 in a wheelchair having difficulties standing up and not being able to walk. DBS was OFF when the video was recorded. Video 2 (P1 post-LCIG): freezing of gait in the ON state in patient 1 after treatment with levodopa/carbidopa intestinal gel. This clip shows the improvement in FOG-ON after treatment with levodopa/carbidopa gel. The patient stands up at first attempt and demonstrates mild freezing when beginning to walk and turning. DBS was OFF when the video was recorded. Video 3 (P3 pre-LCIG): freezing of gait in the ON state in patient 3 before treatment with levodopa/carbidopa intestinal gel. This clip shows patient 3 being unable to rise from the chair and walk without assistance. Severe freezing and generalised dyskinesias are shown. DBS was OFF when the video was recorded. Video 4 (P3 post-LCIG): freezing of gait in the ON state in patient 3 after treatment with levodopa/carbidopa intestinal gel. This clip shows the improvement in FOG-ON after treatment with levodopa/carbidopa gel. The patient is able to rise from the chair with arms-crossed and walks independently with only mild freezing when beginning to walk and turning. DBS was OFF when the video was recorded.
